# Development and Validation of an Immunoassay for Quantification of Topoisomerase I in Solid Tumor Tissues

**DOI:** 10.1371/journal.pone.0050494

**Published:** 2012-12-28

**Authors:** Thomas D. Pfister, Melinda Hollingshead, Robert J. Kinders, Yiping Zhang, Yvonne A. Evrard, Jiuping Ji, Sonny A. Khin, Suzanne Borgel, Howard Stotler, John Carter, Raymond Divelbiss, Shivaani Kummar, Yves Pommier, Ralph E. Parchment, Joseph E. Tomaszewski, James H. Doroshow

**Affiliations:** 1 Laboratory of Human Toxicology and Pharmacology, Applied/Developmental Research Support Directorate, SAIC-Frederick, Inc., Frederick National Laboratory for Cancer Research, Frederick, Maryland, United States of America; 2 Biological Testing Branch, Developmental Therapeutics Program, Frederick National Laboratory for Cancer Research, Frederick, Maryland, United States of America; 3 National Clinical Target Validation Laboratory, SAIC-Frederick, Inc., Frederick National Laboratory for Cancer Research, Frederick, Maryland, United States of America; 4 Applied/Developmental Research Support Directorate, SAIC-Frederick, Inc., Frederick National Laboratory for Cancer Research, Frederick, Maryland, United States of America; 5 Division of Cancer Treatment and Diagnosis, Center for Cancer Research, National Cancer Institute, Bethesda, Maryland, United States of America; 6 Laboratory of Molecular Pharmacology, Center for Cancer Research, National Cancer Institute, Bethesda, Maryland, United States of America; University of California, San Francisco, United States of America

## Abstract

**Background:**

Topoisomerase I (Top1) is a proven target for cancer therapeutics. Recent data from the Fluorouracil, Oxaliplatin, CPT-11: Use and Sequencing (FOCUS) trial demonstrated that nuclear staining of Top1 correlates with chemotherapeutic efficacy. Such a correlation may help identify patients likely to respond to Top1 inhibitors and illuminate their mechanism of action. Cellular response to Top1 inhibitors is complex, but Top1 target engagement is a necessary first step in this process. This paper reports the development and validation of a quantitative immunoassay for Top1 in tumors.

**Methodology/Principal Findings:**

We have developed and validated a two-site enzyme chemiluminescent immunoassay for quantifying Top1 levels in tumor biopsies. Analytical validation of the assay established the inter-day coefficient of variation at 9.3%±3.4% and a 96.5%±7.3% assay accuracy. Preclinical fit-for-purpose modeling of topotecan time- and dose-effects was performed using topotecan-responsive and -nonresponsive xenografts in athymic nude mice. Higher baseline levels of Top1 were observed in topotecan-responsive than -nonresponsive tumors. Top1 levels reached a maximal decrease 4 to 7 hours following treatment of engrafted mice with topotecan and the indenoisoquinoline NSC 724998.

**Conclusions/Significance:**

Our analysis of Top1 levels in control and treated tumors supports the previously proposed mechanism of action for Top1 inhibitor efficacy, wherein higher baseline Top1 levels lead to formation of more covalent-complex-dependent double-strand break damage and, ultimately, cell death. In contrast, xenografts with lower baseline Top1 levels accumulate fewer double-stand breaks, and may be more resistant to Top1 inhibitors. Our results support further investigation into the use of Top1 levels in tumors as a potential predictive biomarker. The Top1 immunoassay described in this paper has been incorporated into a Phase I clinical trial at the National Cancer Institute to assess pharmacodynamic response in tumor biopsies and determine whether baseline Top1 levels are predictive of response to indenoisoquinoline Top1 inhibitors.

## Introduction

Drugs targeting topoisomerase I (Top1) are used in a number of cancer chemotherapy regimens [1]–[5]. Several third-generation Top1 inhibitors are in development [5], [6], including the indenoisoquinolines NSC 743400 (LMP400; HCl salt of NSC 724998) and NSC 725776 (LMP776) which are currently undergoing clinical evaluation at the National Cancer Institute (NCI; ClincalTrials.gov NCT01245192). Recent data from the Fluorouracil, Oxaliplatin, CPT-11: Use and Sequencing (FOCUS) trial demonstrated that nuclear staining of Top1 correlates with the chemotherapeutic efficacy of the Top1 inhibitor irinotecan [7]. However, results from the CApecitabine, IRinotecan, Oxaliplatin (CAIRO) study [8], [9], which also used immunohistochemistry to assess Top1 levels, were inconsistent with the FOCUS trial findings. The immunohistochemistry method is more qualitative and susceptible to sampling variability since typically only a 5 to 7 µm tissue section is analyzed at an area of interest selected by an individual pathologist. In addition, these results do not always correlate with each other or with immunoassay data [10], [11]. These contradictory findings support the need for a more quantitative measurement of Top1 levels in larger specimen samples. Another study using colon cancer cell lines found little correlation between total Top1 levels (as assessed by Western blotting) and efficacy, and concluded that the extent of Top1 cleavage complex (Top1cc) formation may be more informative [12]. It is worth noting that the same factors limiting the correlation of total Top1 measurement to clinical efficacy also apply to measurement of the Top1cc. Top1 levels have been shown to decrease in response to camptothecins via ubiquitylation and proteasome-dependent degradation following trapping of the Top1cc [13]–[21]; thus, decreases in Top1 levels after treatment may also serve as a biomarker of target engagement by compounds binding Top1, or as a biomarker for resistance to Top1 inhibitors.

Topotecan and irinotecan are two currently used Top1 inhibitors that provide an effective palliative regimen for patients with colorectal, small-cell lung, and ovarian cancer. Both drugs are water-soluble derivatives of camptothecin, but are subject to hydrolysis of the alpha-hydrolactone E-ring [22]; an intact E-ring is necessary to trap the Top1 cleavage complex (Top1cc) on DNA [23]. These drugs are also substrates for multidrug-resistance efflux pumps [23]. Several laboratories have synthesized either more stable derivatives of camptothecin or non-camptothecin Top1 inhibitors, such as indolocarbazole, phenanthridine, and indenoisoquinoline derivatives [2], [5]. Currently, the indolocarbazoles edotecarin and NB-506 are in Phase II clinical trials, but these agents may have off-target effects [5], [24]. The phenanthridine derivative ARC-111 has been shown to be more effective than camptothecin in certain tumor models [25], [26]. Indenoisoquinoline compounds have greater chemical stability and form more stable Top1cc than camptothecin [27], and certain indenoisoquinolines (including NSC 724998) are not substrates for the ABC multidrug resistance transport pumps [19].

We have developed and validated several new pharmacodynamic assays in support of clinical trials at the NCI. Our criteria for validation of clinical assays in preclinical models include replicating the sample collection, handling, and analytic procedures that will be used in the clinical setting. We have previously reported on two validated biomarker assays that are currently being used for correlative studies in the clinical trial of indenoisoquinolines NSC 743400 and NSC 725776: a quantitative immunofluorescence assay for measuring histone H2AX phosphorylated at serine 139 (γH2AX) in tumor biopsies, and an assay to detect γH2AX in circulating tumor cells using the CellSearch™ platform [28]–[30]. Increased γH2AX levels are a downstream response to DNA double-stand break (DSB) damage [31] formed by the accumulation of Top1cc on DNA which then becomes “trapped” by Top1 inhibitors [2], [18], [32]. Degradation of Top1cc by the ubiquitin-proteasome pathway may be required, or at least facilitate the formation of DSBs and result in increased levels of γH2AX [18]. Desai et al. have also reported that cells deficient in Top1 degradation may be more sensitive to Top1 inhibitors [13].

As new Top1 inhibitors progress toward clinical evaluation, quantitative methods for measuring Top1 levels directly in solid tumors could be of substantial value for evaluating whether to select patients for indenoisoquinoline therapy based on pre-treatment Top1 levels, or stratifying responding and non-responding patients at the drug target level. Of consideration is that the cellular and molecular pharmacology of Top1 inhibitors is complex, and while Top1 target engagement is a necessary first step in this process, it may not be a predictor for tumor response. Therefore, we set out to establish the utility of monitoring pharmacodynamic response to Top1 inhibitors with a validated assay for Top1 levels as a measure of target engagement, and to determine the relationship between pre- and post-treatment Top1 levels in experimental models. We have reported on the development and validation of an enzyme-linked immunosorbent assay (ELISA) for quantifying Top1 levels in cancer cell lines [33]. In the current study, we have validated the Top1 immunoassay for use with solid tumor extracts and report preclinical modeling with a non-camptothecin indenoisoquinoline Top1 inhibitor and topotecan against xenograft models. As part of method validation, we also demonstrated the ability to measure baseline Top1 levels in 18-gauge needle tumor biopsies collected from patients in clinical trials at the NCI following standard operating procedures established for the clinical poly(ADP-ribose) immunoassay [34].

## Results

### Assay validation

**Figure 1:**
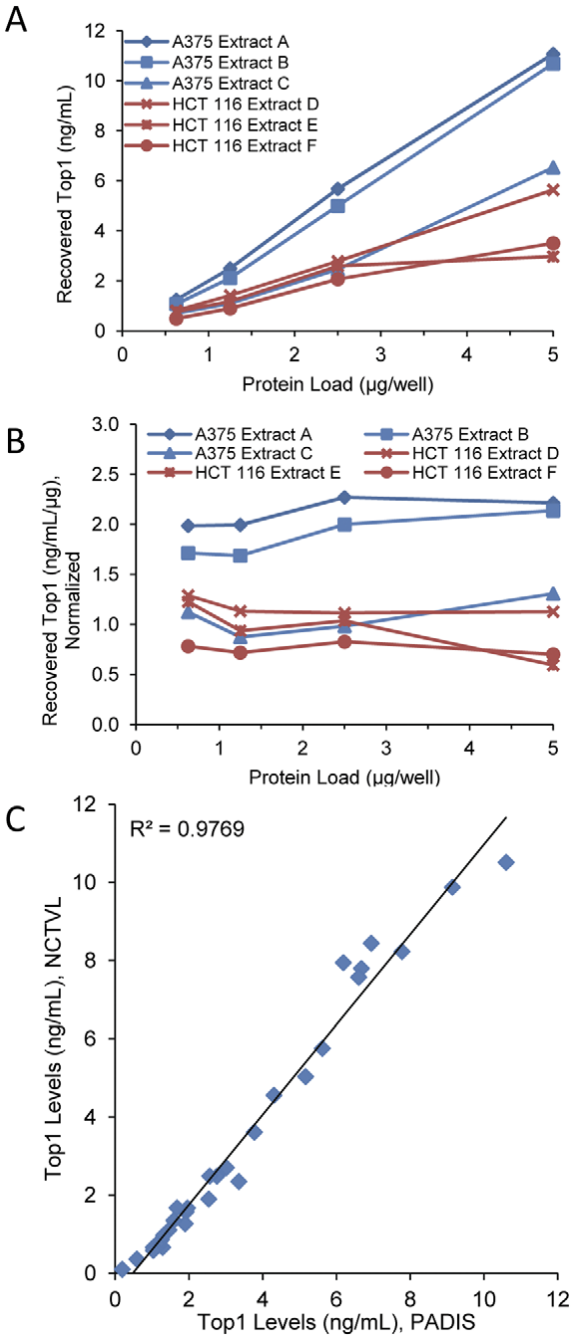
Validation of assay performance. (A) Top1 levels were measured in serial dilutions of three A375 and three HCT 116 xenograft extract samples using the Top1 immunoassay. (B) Total Top1 levels from the diluted extracts in panel A normalized to 1 μg/mL. (C) Comparison of Top1 levels in 24 matched samples and 6 control extracts, measured by two independent laboratories, the Pharmacodynamic Assay Development and Implementation Section (PADIS) laboratory and the National Clinical Target Validation Laboratory (NCTVL). Sample dilution and analysis were performed independently by both laboratories and Top1 levels were compared across sites.

We designed an assay to measure total Top1 in cell extracts, both free and DNA bound. Analytical validation was carried out to establish assay accuracy and precision. During sample collection and processing, samples were kept on ice at all times to prevent loss of Top1 signal; experiments performed during early assay development had demonstrated that boiling Top1 standards resulted in a loss of signal (unpublished data). In addition, when samples were stored as diluted extracts (0.1 mg/mL), Top1 tended to be degraded compared to fresh extracts or extracts stored at concentrations ≥1.0 mg/mL (unpublished data). Accuracy was evaluated by spike/recovery of pure recombinant Top1 (rTop1) standards in tumor biopsy extracts and by analysis of dilution linearity of intrinsic Top1 in xenograft samples. Dilution linearity was demonstrated by measuring intrinsic Top1 levels in three A375 and three HCT 116 xenograft extracts; Top1 levels reported by the assay showed acceptable dilution linearity over the protein load range (Figures 1A and 1B). An antigen spike-recovery experiment was used to determine assay accuracy. Pure rTop1 standards were spiked into A375 extracts at three different protein loads and Top1 recovery was measured; recovery of antigen ranged from 81% to 105% with a mean ± standard deviation (SD) of 96.5%±7.3% (Table 1).

**Table 1:** Percent recovery of rTop1 standards spiked into A375 xenograft extracts at three different protein loads.

rTop1 standard	% Recovery by protein load		
(ng/mL)	1.0 μg Protein load	0.5 μg Protein load	0. 25 μg Protein load
0.391	101	101	97
0.781	99	101	81
1.563	101	105	84
3.125	99	101	87
6.25	94	104	93
**Mean recovery ± SD**	98.8±2.7	102.3±2.1	89.6±6.6

Abbreviations: SD =  standard deviation.

Assay precision was assessed by inter-day and intra-assay variability of results. Inter-day performance was determined by measuring Top1 levels in HCT 116 xenograft samples over three days by a single operator. The coefficient of variation (CV) for inter-day performance ranged from 2.0% to 17.8% with a mean ± SD of 9.3%±3.4% (Table 2). Intra-assay performance was calculated using the same data and CVs ranged from 0.2% to 11.5%. Inter-laboratory reproducibility was determined by measuring Top1 levels in 24 matched HCT 116 xenograft biopsy extract samples and matched controls in two different laboratories, National Clinical Target Validation Laboratory (NCTVL) and Pharmacodynamic Assay Development and Implementation Section (PADIS). Analysis carried out by separate operators in each laboratory showed a strong correlation between the two sites (R^2^ = 0.98; Figure 1C).

**Table 2:** Inter-day and intra-assay performance of the Top1 immunoassay.

	Protein load	Inter-day performance (Top1 levels, ng/mL)						Inter-day	Intra-assay CV%		
	(µg/well)	Day 1	Day 2	Day 3	Mean	±	SD	CV%	Day 1	Day 2	Day 3
HCT 116-1	5	3.40	4.35	3.53	3.76	±	0.52	13.8	1.5	0.5	0.9
	3.75	2.46	2.69	2.73	2.62	±	0.15	5.6	3.1	2.7	6.3
	2.5	1.48	1.64	1.72	1.61	±	0.12	7.5	1.6	0.5	3.5
	1.25	0.63	0.66	0.77	0.69	±	0.07	10.2	1.1	0.9	1.0
HCT 116-2	5	8.83	10.29	10.51	9.88	±	0.91	9.2	0.5	0.2	2.3
	3.75	6.83	8.35	8.21	7.8	±	0.84	10.7	2.7	4.0	1.4
	2.5	4.19	5.11	4.42	4.57	±	0.48	10.5	3.2	0	2.1
	1.25	1.63	1.79	1.81	1.74	±	0.1	5.5	0.4	3.2	0.5
HCT 116-3	5	2.15	2.72	2.44	2.44	±	0.28	11.6	3.4	1.8	6.5
	3.75	1.77	2.10	1.99	1.95	±	0.17	8.7	2.3	1.4	4.8
	2.5	1.19	1.34	1.42	1.31	±	0.12	8.9	2.3	1.4	1.8
	1.25	0.64	0.65	0.78	0.69	±	0.08	10.9	0.5	0.4	0.7
HCT 116-4	5	7.52	9.29	8.52	8.44	±	0.89	10.5	0.2	0.3	2.0
	3.75	5.61	6.22	5.60	5.81	±	0.36	6.1	1.6	1.2	1.8
	2.5	2.94	2.96	2.79	2.89	±	0.09	3.1	4.3	3.9	1.1
	1.25	0.76	0.70	0.85	0.77	±	0.08	9.9	1.6	0.7	1.6
HCT 116-5	5	1.29	1.38	1.52	1.40	±	0.12	8.3	3.3	2.9	5.9
	3.75	1.05	1.1	1.27	1.14	±	0.11	10	4.6	0.7	2.7
	2.5	0.82	0.83	0.98	0.88	±	0.09	10.5	0.8	2.0	1.8
	1.25	0.34	0.34	0.44	0.38	±	0.05	14.4	2.6	0.6	2.3
HCT 116-6	5	6.85	8.53	8.45	7.95	±	0.95	11.9	0.2	0.4	4.2
	3.75	4.25	5.39	4.87	4.84	±	0.57	11.8	0.8	0.8	1.1
	2.5	2.30	2.42	2.58	2.43	±	0.14	5.7	11.5	0.4	5.1
	1.25	0.52	0.60	0.74	0.62	±	0.11	17.8	1.2	0.4	2.8
A375 controls	High	10.02	10.95	10.57	10.52	±	0.47	4.4	1.8	4.5	2.6
	Mid	6.52	7.88	7.47	7.29	±	0.70	9.5	1.9	0.7	3.8
	Low	1.70	1.75	1.68	1.71	±	0.03	2.0	9.1	8.9	0.9
MCF7 controls	High	6.67	8.33	7.72	7.57	±	0.84	11.1	3.3	2.0	2.0
	Mid	1.00	1.04	1.14	1.06	±	0.07	6.7	3.3	2.8	5.7
	Low	< LLQ	0.11	0.13	0.11	±	0.01	13.1	1.4	1.7	2.2

Abbreviations: CV%, percent coefficient of variation; LLQ, lower limit of quantitation; SD, standard deviation.

### Drug efficacy studies

Efficacy of the indenoisoquinoline NSC 724998 compared to topotecan was tested against A375, HCT 116, Colo829, and SK-MEL-28 xenografts in athymic nude mice. Top1 inhibitors were administered to xenograft-bearing mice once daily for 5 days as described in the methods. Tumor growth data for topotecan and NSC 724988 in the A375 model have previously been reported [30]. A375 and HCT 116 xenografts in mice had both a log cell kill and a delay in tumor growth (measured by changes in median tumor volume) following topotecan treatment in comparison to the vehicle controls; these results indicated that the A375 and HCT 116 xenograft models were topotecan-responsive (Table 3). SK-MEL-28 xenografts were considered nonresponsive as no tumor growth inhibition was observed at any topotecan dose or regimen tested, and Colo829 xenografts were moderately responsive (Table 4).

**Table 3:** Tumor response and biomarkers for Top1 inhibitors in topotecan-responsive xenograft models.

			Tumor growth inhibition		
	No. of mice^a^	Drug-related deaths^b^	Maximum % mean body weight loss (d)	Growth delay % (T-C)/C	Net log cell kill
**A375 Xenografts** c					
Topotecan, IP					
4.7 mg/kg QD×5	8	2	28.4 (15)	143	1.8
1.5 mg/kg QD×5	8	0	6.4 (12)	54	0.3
NSC 724998, IV					
16.0 mg/kg QD×5	8	0	7.2 (12)	259	0.8
12.0 mg/kg QD×5	8	0	7.1 (12)	116	1.4
8.0 mg/kg QD×5	8	0	3.6 (12)	68	0.6
4.0 mg/kg QD×5	8	0	3.2 (12)	28	−0.1
**HCT 116 Xenografts**					
Topotecan, IP					
4.0 mg/kg QD×5	8	0	13.7 (14)	79	0.4
2.0 mg/kg QD×5	8	0	16.4 (21)	45	0.1
1.0 mg/kg QD×5	8	0	18.4 (24)	43	0.0
NSC 724998, IV					
25.0 mg/kg QD×5	8	0	11.4 (52)	67	1.0
16.75 mg/kg D×5	8	0	11.9 (43)	57	0.8
11.2 mg/kg QD×5	7	0	0.0 (NA)	47	0.6

Abbreviations: C, control group; d, day; IP, intraperitoneal; IV, intravenous; NA, not applicable; ND, not determined QD×5, treated for 5 sequential days at designated dose; T, treated group.

a . No mice were tumor-free by study day 70.

b . A death is considered treatment-related if the animal dies within 15 days of the last treatment and either the tumor weight is less than the lethal burden in the control mice or its net body weight loss at death is 20% greater than the mean net weight change in the control animals at death or sacrifice.

c . Data from topotecan and NSC 724998 treated A375 xenografts in athymic nude mice were previously published by our group [30].

**Table 4:** Tumor response and biomarkers for Top1 inhibitors in topotecan-nonresponsive and moderately responsive xenograft models.

			Tumor growth inhibition		
	No. of mice^a^	Drug-related deaths^b^	Maximum % mean body weight loss (d)	Growth delay % (T-C)/C	Net log cell kill
**Colo829 Xenografts**					
Topotecan, IP					
4.0 mg/kg QD×5	13	0	11.0 (34)	6	−0.1
1.0 mg/kg QD×5	13	0	9.2 (34)	−15	−0.4
NSC 724998, IV	ND				
**SK-MEL-28 Xenografts**					
Topotecan, IP					
4.0 mg/kg QD×5	12	0	15.8 (22)	12	0.0
NSC 724998, IV					
16.0 mg/kg QD×5	12	0	5.6 (18)	7	0.0

Abbreviations: C, control group; d, day; IP, intraperitoneal; IV, intravenous; NA, not applicable; ND, not determined QD×5, treated for 5 sequential days at designated dose; T, treated group.

a . No mice were tumor-free by study day 70.

b . A death is considered treatment-related if the animal dies within 15 days of the last treatment and either the tumor weight is less than the lethal burden in the control mice or its net body weight loss at death is 20% greater than the mean net weight change in the control animals at death or sacrifice.

### Baseline Top1 levels in topotecan-responsive and -nonresponsive xenograft models

**Figure 2:**
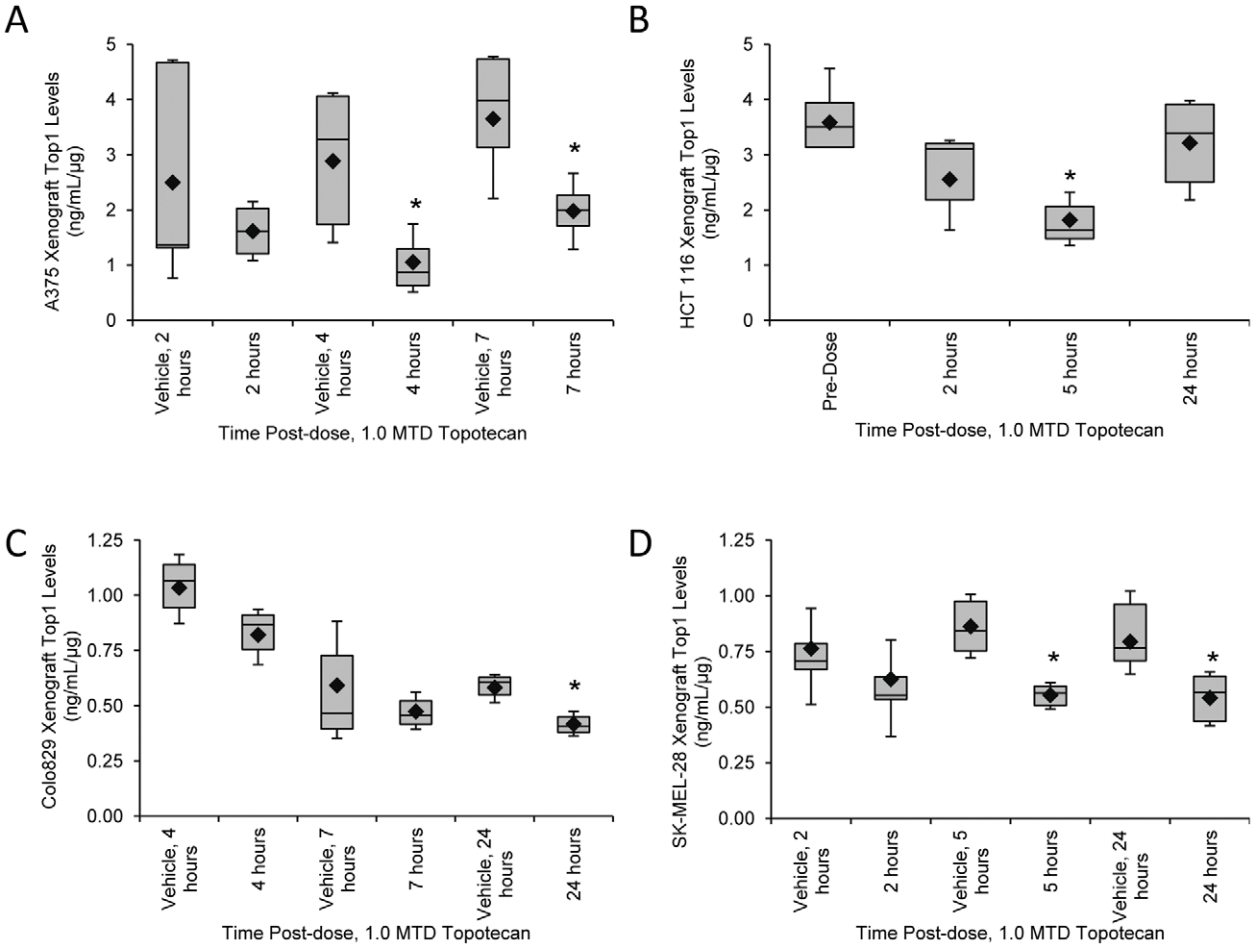
Top1 levels in topotecan-responsive and -nonresponsive xenograft models. Mice treated with water vehicle or single dose 1.0 MTD topotecan for the designated times were assessed for Top1 levels using the validated immunoassay. (A) Mice bearing A375, topotecan-responsive xenografts were collected 2, 4, or 7 hours after treatment (n = 4/cohort). (B) HCT 116 colon cancer xenografts were collected 2, 5, and 24 hours after treatment and compared to baseline (0 h, no vehicle). n = 3–6 topotecan-treated mice/cohort and n = 12 in the 0 h cohort. (C) Colo829 xenografts were collected 4, 7, or 24 hours after treatment (n = 3/cohort). (D) SK-MEL-28 xenografts were collected 2, 5, or 24 hours after treatment (n = 6/cohort). Box plots represent interquartile range, 10^th^ and 90^th^ percentile whiskers, and median Top1 levels; diamonds represent group mean. Asterisks (*), treatment cohort mean was statistically different from the vehicle mean at same time point (or baseline in the HCT 116 cohorts), with a significance level of P≤0.05, as determined by Student's one-tailed *t*-test.

Top1 levels in all xenografts from A375 engrafted untreated and vehicle control mice varied by 40% (n = 25, Table 5) with 15 of 25 mice in the control groups having Top1 levels within 1 SD of the mean. Top1 levels in the vehicle group ranged from 0.43 to 4.75 ng/mL Top1 per 1 μg protein (ng/mL/μg; Figure 2A). This variation was much greater than the expected percent CV for intra- and inter-assay performance and likely represents biological differences in the samples, possibly due to specimen collection and handling procedures prior to assaying. Baseline Top1 levels in HCT 116 xenografts ranged from 2.68 to 4.65 ng/mL/μg (Figure 2B); variation in all untreated and vehicle-treated HCT 116 xenografts was 20% (n = 15; Table 5). A 25% variation in Top1 levels from the mean was seen in all vehicle-treated Colo829 (n = 12) and 33% in all vehicle-treated SK-MEL-28 (n = 18). Top1 levels of vehicle-treated xenografts ranged from 0.32 to 1.21 ng/mL/μg in Colo829 xenografts and 0.49 to 1.08 ng/mL/μg in SK-MEL-28 xenografts (Figure 2C and 2D). Of note, the baseline levels of Top1 differed in the four different xenograft models with higher baseline Top1 levels present in the topotecan-responsive A375 and HCT 116 xenografts, 3.01±1.58 ng/mL/μg and 3.58±0.65 ng/mL/μg, respectively, and lower levels in the moderately responsive Colo829 and nonresponsive SK-MEL-28 models, 0.74±0.3 ng/mL/μg and 0.81±0.16 ng/mL/μg, respectively. Top1 levels in the Colo829 and SK-MEL-28 models were significantly lower than the topotecan-responsive models by Student's unpaired two-tailed *t*-test (P≤0.0005), but not from each other.

**Table 5:** Biological variation observed in baseline Top1 levels in xenograft models and patient samples.

	No. of samples	Top1 level, ng/mL/μg (mean ± SD)	No. samples within 1 SD of the mean	Percent variation (%)
**Xenografts** a				
A375	25	2.87±1.25	15	40
HCT 116	15	3.42±0.82	12	20
Colo829	12	1.07±0.68	9	25
SK-MEL-28	18	0.81±0.17	12	33
**Patient Samples**				
Tumor biopsy^b^	6	1.53±1.38	4	33
PBMCs^c^	14	34.5±28.7 ng/mL per 1×10^7^cells	12	15

Abbreviations: PBMCs, peripheral blood mononuclear cells; SD, standard deviation.

a . Xenograft data include Top1 levels measured in untreated and vehicle-treated (water) A375 and HCT 116 xenograft bearing mice and vehicle-treated (water) Colo829 and SK-MEL-28 mice from all experiments.

b . Top1 levels measured in tumor biopsies from patients at baseline. Top1 levels expressed as an average of all measurements above the LLQ; each patient had Top1 levels above the LLQ for at least 2 of 3 protein loads.

c . Top1 levels measured in PBMC samples from patients at baseline. Top1 levels expressed as an average of all measurements above the LLQ. In one patient, the Top1 levels were below the LLQ, in this case the LLQ was used as the Top1 measurement.

### Comparison of Top1 levels following topotecan treatment in topotecan-responsive and -nonresponsive xenografts models

Effects of topotecan treatment were compared in the topotecan-responsive and -nonresponsive xenograft models at baseline (untreated or vehicle treated) and varying time points during the first 24 hours after a single dose of 1.0 maximum tolerated dose (MTD) topotecan. Top1 levels in the A375 xenograft model decreased 63% at 4 hours and 46% at 7 hours compared to matched vehicle cohorts following topotecan treatment; these decreases were significant by Student's one-tailed *t*-test (P = 0.02 and 0.03, respectively; Figure 2A). Mean Top1 levels in untreated mice bearing HCT 116 xenografts were 3.59±0.65 ng/mL/μg and decreased 49% to 1.82±0.6 ng/mL/μg 5 hours after topotecan treatment (P = 0.0009; Figure 2B). While a trend for decreasing Top1 levels over time was seen in the moderately topotecan-responsive Colo829 xenograft model, the matched vehicle controls also had decreasing Top1 levels (Figure 2C). Only at 24 hours following topotecan treatment was a statistically significant decrease in Top1 observed (P = 0.03). The topotecan-resistant SK-MEL-28 model had a less pronounced (Figure 2D), but yet significant decrease in Top1 levels 5 hours (36% decrease) and 24 hours (32% decrease) after topotecan administration (P = 0.0002 and 0.01, respectively; Figure 2D).

### Dose response over time in the A375 topotecan-responsive xenograft model

**Figure 3:**
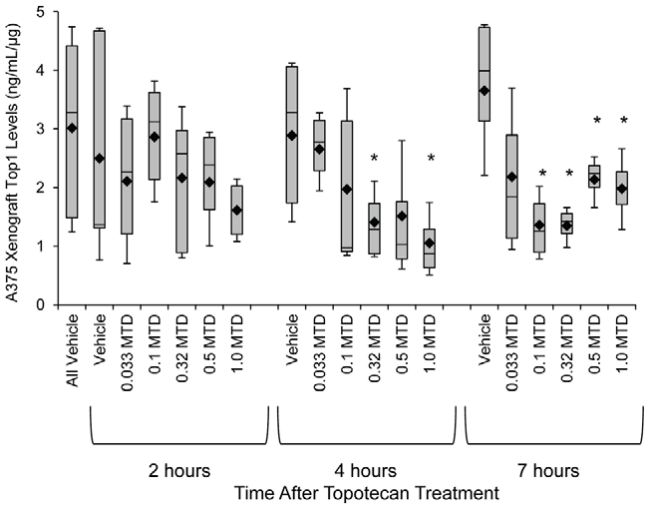
Dose-dependent decrease in Top1 levels in A375 xenografts. Tumor biopsies were collected from A375 xenografts 2, 4, and 7 hours after a single dose of 0.033 to 1.0 MTD topotecan or water vehicle control (n = 4/cohort). Box plots represent interquartile range, 10^th^ and 90^th^ percentile whiskers, and median Top1 levels; diamonds represent group mean. Asterisks (*), treatment cohort mean was statistically different from vehicle mean at same time point, with a significance level of P≤0.05, as determined by Student's one-tailed *t*-test.

During validation of our γH2AX immunofluorescence assay, xenograft modeling was used to determine the optimal timing for biomarker assessment following Top1 inhibitor treatment [30]. We replicated this analysis for the Top1 immunoassay in mice bearing A375 xenografts treated with single dose 0.033 to 1.0 MTD topotecan for 2, 4, or 7 hours and compared to matched vehicle control groups. Decreases in Top1 levels were not significant 2 hours after topotecan treatment at any dose level when compared to matched vehicle controls (Figure 3, left). In the 4-hour post-dose treatment groups, Top1 levels decreased by 51% following 0.32 MTD topotecan treatment (P = 0.03), 48% following 0.5 MTD treatment (P = 0.09), and 63% in the 1.0 MTD group (P = 0.02) when compared to the 4 hours vehicle control. Although not all 4-hour post-dose groups had significantly reduced Top1 levels compared to vehicle, regression analysis of the Top1 levels over increasing topotecan dose showed a good correlation with an R^2^ of 0.72. By 7 hours following topotecan treatment, all mouse groups except those treated with the lowest topotecan dose had significantly decreased Top1 levels compared to the vehicle group (P≤0.05) with a 63% reduction in Top1 seen in the 0.1 and 0.32 MTD dosing groups. Though there was a high degree of variation seen in the vehicle control groups collected at different time points (Top1 range, 0.4–4.81 ng/mL/μg), there was no statistical difference between them.

### Top1 levels in xenografts from topotecan- or indenoisoquinoline-treated mice

**Figure 4:**
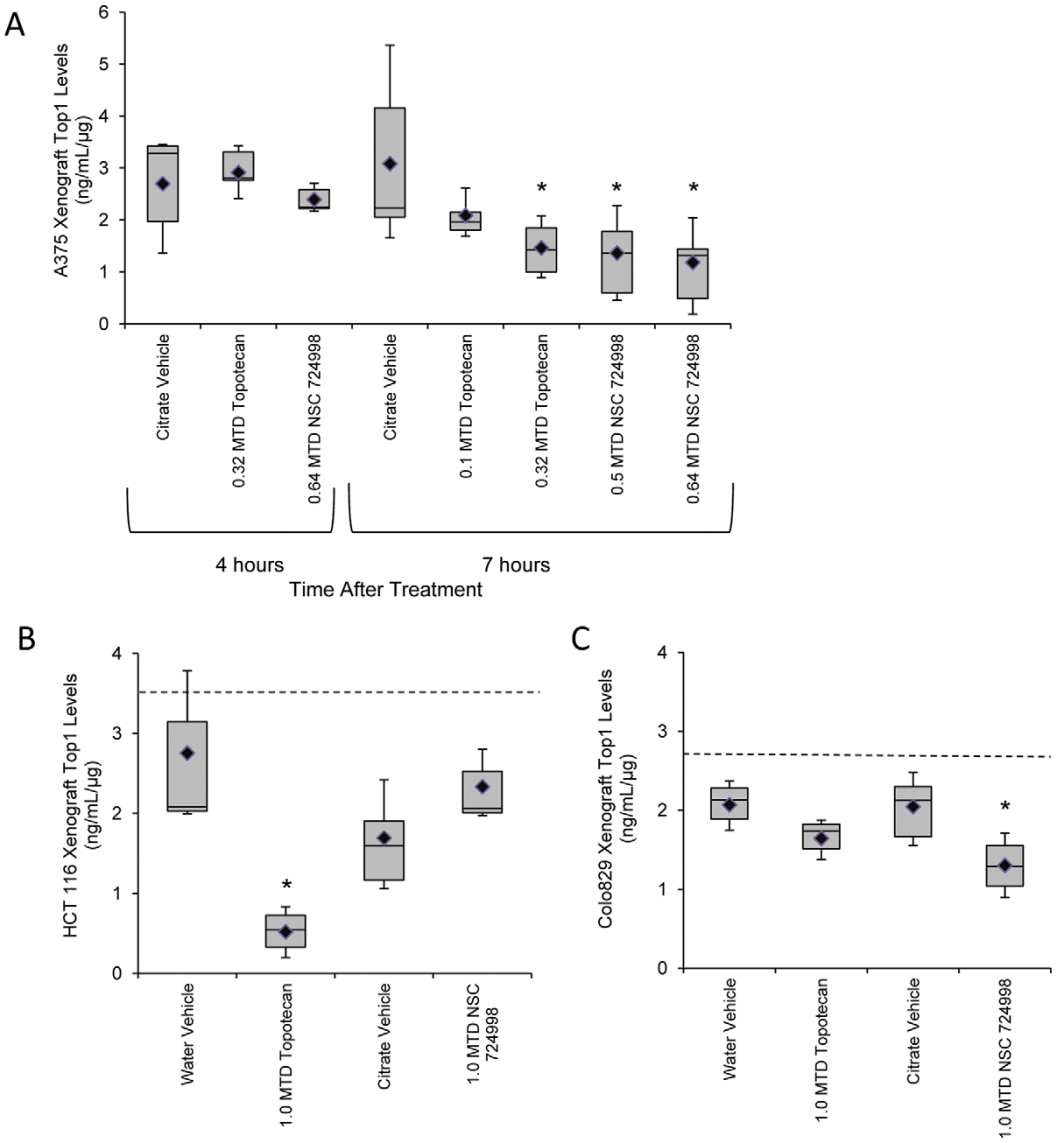
Top1 levels measured in xenografts treated with topotecan compared to two indenoisoquinoline topoisomerase inhibitors. (A) Top1 levels in A375 xenografts collected 4 or 7 hours following single dose treatment (MTD indicated) with topotecan or the indenoisoquinoline NSC 724998 (n = 5–6 mice/cohort). (B) HCT 116 xenografts and (C) Colo829 xenografts collected 4 hours after mice were treated with the indicated doses and drugs. (n = 3–6 mice/cohort; NSC 724998 had 2 mice/cohort). Box plots represent interquartile range, 10^th^ and 90^th^ percentile whiskers, and median Top1 levels; diamonds represent group mean. Dashed lines in panels B and C indicate the mean Top1 levels in a matched untreated cohort (0 h) for these xenograft models. Asterisks (*), treatment cohort mean was statistically different from vehicle mean at same time point, with a significance level of P≤0.05, as determined by Student's one-tailed *t*-test.

Mice bearing A375 xenografts were treated with increasing doses of topotecan or NSC 724998, and Top1 levels were measured at 4 or 7 hours following treatment (Figure 4A). No significant decrease in Top1 levels was observed 4 hours after 0.32 MTD topotecan or 0.64 MTD NSC 724998 treatment compared to citrate vehicle controls. By 7 hours post-dose, a significant decrease in Top1 levels was seen in the 0.32 MTD topotecan (P = 0.03), 0.5 MTD NSC 724998 (P = 0.03), and 0.64 MTD NSC 724998 (P = 0.02) treatment cohorts. The 0.1 MTD topotecan-treated cohort was not statistically different from matched citrate vehicle controls. The timing of maximal Top1 depletion matched that seen with earlier topotecan experiments in the A375 xenograft model.

HCT 116 and Colo829 xenograft-bearing mice were treated with single dose 1.0 MTD topotecan or NSC 724998 and samples were collected for Top1 evaluation 4 hours later. Top1 levels were compared to matched vehicle controls. In the HCT 116 xenografts, only 1.0 MTD topotecan treated mice had a significant decrease in Top1 levels versus its matched vehicle control (P = 0.02; Figure 4B). Mean Top1 levels for HCT 116 xenograft bearing mice treated with NSC 724998 were not statistically different from their corresponding citrate vehicle group. As seen previously, mean Top1 levels in Colo829 xenografts decreased following 1.0 MTD topotecan treatment, though this change was not significant (Figure 4C). In contrast, the 1.0 MTD NSC 724998 treated Colo829 cohort had significantly decreased Top1 levels compared to matched vehicle controls (P = 0.03).

In both the HCT 116 and Colo829 models, an untreated cohort was examined in parallel with the vehicle control and drug treated cohorts. Top1 levels in HCT 116 xenografts from untreated mice averaged 3.53±1.4 ng/mL/μg and in Colo829 averaged 2.73±0.12 ng/mL/μg (mean ± SD, dashed line in Figures 4B and 4C). While Top1 levels in the water vehicle HCT 116 cohort collected 4 hours after treatment were not statistically different from the untreated cohort, the citrate vehicle control group had significantly lower Top1 levels than the untreated group (P≤0.05). In the Colo829 xenograft samples, both the water vehicle and citrate vehicle groups had statistically lower Top1 levels than the untreated group (P≤0.05).

### Baseline Top1 levels in patient tumors and PBMC samples

**Figure 5:**
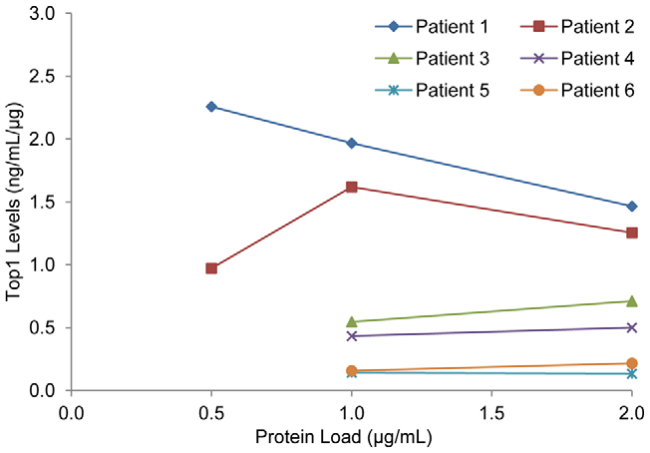
Baseline Top1 levels in clinical biopsy samples. Top1 levels measured in extracts prepared from pre-treatment tumor needle biopsies from 6 patients enrolled in NCI clinical trials. Top1 levels were assayed using three different protein loads for each extract. Top1 levels in biopsy extracts for the 0.5 μg/mL protein load of patients 3 to 6 were below the LLQ of the assay.

Determination of Top1 baseline levels in tumor biopsy extracts from six patients enrolled in clinical trials at the NCI showed 4 of 6 patients had Top1 levels within 1 SD of the mean (Table 5). Top1 concentrations for individual patients were determined by averaging three different protein loads for each tumor extract. Only values above the lower limit of quantitation (LLQ) were reported; patient baseline tumor biopsy Top1 levels ranged from 0.37 to 3.79 ng/mL/μg biopsy protein. During Top1 immunoassay analysis as part of fit-for-purpose testing, patient Top1 levels are measured in triplicate at three protein loads (Figure 5) to ensure that calculation of Top1 levels in a patient sample is determined within the linear range of the sample. In the six patient samples analyzed, based on the calculated total baseline Top1 levels at the highest protein load (2 µg/well), the assay could have detected a 90% reduction in Top1 levels in 4 of the 6 (66%) patients, a 70% reduction in 5 of the 6 patients (83%), and a 60% reduction in all 6 patients. Top1 levels in peripheral blood mononuclear cells (PBMCs) had a 15% variation with Top1 ranging from 9.9 to 103.8 ng/mL/1×10^7^cells (Table 5). Only 1 of 15 PBMC samples analyzed read below the LLQ and the fraction of specimens in which a 70% or greater reduction in PBMC levels could be measured was 53%, while a 50% reduction could be measured in 73% of PBMC specimens.

## Discussion

Numerous xenograft studies have demonstrated that treatment with Top1 inhibitors results in tumor regression [24], [35]–[37]; however, to our knowledge, none of these studies have measured target engagement by the test drugs. Our group recently reported dose- and time-dependent changes in γH2AX levels in tissue sections and blood from xenograft-bearing mice treated with indenoisoquinolines or topotecan [30]. Changes in γH2AX signal following Top1 inhibitor treatment provide a downstream marker for Top1-induced DNA damage. The quantitative immunoassay for Top1 enzyme levels provides a more direct measurement of drug activity on target, and was validated to measure Top1 inhibitor effects in solid tumor extracts. The assay was used for preclinical pharmacodynamic modeling of indenoisoquinoline Top1 inhibitors compared to topotecan.

Assay validation studies indicate that the Top1 immunoassay is suited for preclinical modeling and use in clinical trials for Top1 inhibitors. The assay had good inter-assay precision, acceptable spike recovery, and a good correlation between separate laboratories. During assay development it was noted that in order to prevent loss of Top1 signal, samples had to be kept refrigerated during processing; procedures were modified to maintain samples on ice. Time-course studies of Top1cc by several laboratories indicate that Top1cc reverses at the same time points used with our assay (4–7 hours post-treatment); therefore, heat reversal to release the DNA-bound Top1 was not used [12], [38]–[40]. Additionally, the presence of Top1cc was not expected to affect total Top1 assay readout since both the capture and detection antibodies used in the assay bind both Top1 and Top1cc (data not shown) and sample processing used a Pro 200 homogenizer which shears DNA and should facilitate antibody binding to Top1cc. It is also likely that under our assay conditions, Top1cc are converted to free Top1 as a result of sample dilution since the equilibrium greatly favors the free (dissociated) enzyme in the absence of drug [41]. However, it still remains possible that some Top1 that is associated with DNA as Top1cc may be undetected in our assay; in this case our assay would read this as a decrease in total Top1. Whether we are measuring true Top1 degradation or an apparent decrease due to Top1cc formation, the final assay readout remains a report of engagement of target. Of note, our data show little or no decrease in Top1 levels at 2 hours where Top1cc should be greater, while a decrease is observed at 4 hours when Top1cc should have decreased [41].

### Baseline Top1 levels in topotecan-responsive and -nonresponsive xenograft models

Our data support previous findings that pretreatment levels of Top1 may act as an indicator of responsiveness [7], [13], [42] and appear to be a better indicator than Top1 levels reached during treatment. Topotecan-responsive xenografts (A375 and HCT 116) had higher baseline levels of Top1 in untreated or vehicle treated mice than the topotecan-nonresponsive model (SK-MEL-28). Colo829 xenografts, which exhibited an intermediate response to topotecan, had correspondingly intermediate levels of Top1.

Top1 levels decreased upon treatment with 1.0 MTD topotecan, consistent with reports that Top1 is degraded in response to camptothecin [13]–[18], which shares the E-ring structure and mechanism of inhibition with topotecan. Interestingly, our previously published evaluation of Top1 levels in the NCI 60 cancer cell line panel revealed that HCT 116 had one of the highest Top1 levels, about 9-fold higher than the SK-MEL-28 cell line, which had one of the lowest Top1 levels in the panel [33].

One mechanism of resistance for cells with lower Top1 levels is the formation of fewer Top1cc, resulting in lower accumulation of DNA damage and increasing the likelihood of effective repair [2], [23]. Recent findings by our group show that topotecan-responsive A375 xenografts have much higher levels of γH2AX foci after Top1 inhibitor treatment than topotecan-nonresponsive SK-MEL-28 xenografts supporting a higher frequency of DNA DSB damage in topotecan-responsive tumors [30]. These observations support the hypothesis that the initial Top1 levels are critical for drug response and that baseline levels of Top1 may provide a biomarker for predicting drug response. Analogously, a targeted RNAi approach has demonstrated that suppression of Top1 produces resistance to camptothecin [43], [44]. While potentially a powerful predictive tool, measurement of Top1 levels only provides a metric of target engagement with Top1 inhibitors. The ultimate response to chemotherapy will almost certainly depend on additional factors including DNA repair and apoptotic signaling pathways present in the tumor.

### Reduction of Top1 levels in xenografts from topotecan- or indenoisoquinoline-treated mice

Our study suggests that reduction in Top1 levels could be used as a biomarker for drug engagement of target. Colo829 and HCT 116 xenograft models provide data supporting the link between Top1 levels, decrease in Top1 upon treatment, γH2AX induction (unpublished data) and tumor response. Top1 levels in topotecan-treated A375 xenografts measured using our Top1 immunoassay showed a time- and dose-dependent decrease in response to topotecan treatment. These results parallel our previously reported in vitro results [33] and prior results in a panel of cell lines with varying response to Top1 inhibitors [13]. Maximal Top1 down regulation in responsive xenograft models was observed 4 to 7 hours post-treatment, suggesting that this may be the optimum time window to collect a post-dose tumor biopsy from patients. However, the large variability in Top1 levels in the control animals may complicate its use as a pharmacodynamic marker. The observed variability likely stems from biological variability, as the analytical variability of the assay is low. Substantial effort was made to establish optimal conditions for specimen handling and Top1 recovery efficiency to match those of standard clinical chemistry assays; however, the possibility remains that some variation was due to specimen collection and handling procedures. In addition, assay transfer across laboratories showed a high degree of reproducibility (R^2^ = 0.98), including specimen extraction steps. Using each individual tumor as its own control, as is frequently done in early-stage clinical trials, may minimize the effect of individual differences in baseline Top1 levels.

Overall Top1 levels in untreated HCT 116 xenografts were less variable than in A375 xenografts, which may make HCT 116 a more tractable model for certain preclinical studies of Top1 inhibitors, even though tumor growth inhibition was not as great as in the A375 model. Also, it should be noted that a large degree of variability in Top1 levels was observed in untreated, water vehicle-treated, and citrate vehicle-treated HCT 116 xenografts. Top1 levels decreased significantly with 1.0 MTD topotecan treatment, but were not statistically different from vehicle or 1.0 MTD NSC 724998 treated groups. Additional data (not shown) suggest that Top1 levels in a subset of HCT 116 xenografts 24 hours after 1.0 MTD topotecan and 1.0 MTD NSC 724998 treatment are lower than control animals. This may be due to differences in the baseline levels of Top1 being reflective of two populations of response, or may be due to differences in rates of Top1 synthesis and degradation. The sustained low levels of Top1 in responding models, corresponding to the minimal Top1 levels in the non-responding models, may be critical to understanding the optimal time to re-administer drug.

Even though SK-MEL-28 xenografts were defined as topotecan-nonresponsive by tumor growth inhibition analysis, Top1 levels were significantly decreased at 5 and 24 hours after a single dose of 1.0 MTD topotecan. Western blot data also show Top1 is down regulated in both A375 and SK-MEL-28 cells (Figure S1); thus, Top1 targeting and target engagement are not effective in SK-MEL-28 cells. One explanation could be that the low endogenous levels of Top1 in SK-MEL-28 cells may result in the accumulation of fewer Top1cc, resulting in less DNA damage, and ultimately allowing the cells to survive exposure to Top1 inhibitors. Indeed, biopsies from SK-MEL-28 xenografts display a minimal γH2AX response at 2 and 5 hours after a single dose of 1.0 MTD topotecan compared to biopsies from A375 xenografts, which have a considerable γH2AX response [30].

One difference between responsive and non-responsive tumors may, at least in part, be due to the amount of DNA damage induced in the cells. Top1 inhibitors such as camptothecin and the non-camptothecin indenoisoquinolines share a common mechanism of action, such that they trap the Top1cc by binding at the DNA-Top1 interface [2]. The level of damage induced by these inhibitors will likely depend on not only how much drug binds to the DNA-Top1cc, but also how much target (Top1) is present in the cell to arrest processing and promote DNA DSBs. Cells with low Top1 levels likely do not generate enough DNA damage to induce cell killing; thus, cells expressing low levels of Top1 and/or cells with depleted Top1 protein levels due to Top1 poisoning are resistant because not enough additional DNA damage can be induced to kill the cells. Additional factors such as drug countertransport, driver mutations, tumor-stroma interactions, rate of cell division, DNA repair deficiencies, and apoptotic response [2] may play a role in modulating this effect. An additional factor could be whether the amount of induced DNA damage is greater than can be repaired by the DNA repair pathways. Formation of the Top1cc is known to result in DNA DSBs as DNA or RNA polymerases collide with trapped Top1cc [2], and increased DNA DSBs could ultimately lead to cell death if repair is not successfully completed. Trapping of Top1, ubiquitylation, and degradation by the proteasome [13] can also reduce the amount of Top1 target available to induce further DNA damage. Accordingly, some cells that are deficient in Top1 degradation despite formation of Top1cc, have increased sensitivity to Top1 inhibitors [13].

### Top1 levels in patient tumors and PBMC samples

Baseline Top1 levels in both patient tumor and PBMC samples show similarly large variation as that measured in xenografts. This variation suggests that it may not be possible to measure drug effect based on post-dose Top1 levels alone, but will require a paired pre-dose specimen from the same patient against which target inhibition by the drug can be gauged.

### Summary and conclusions

The development and validation of a quantitative assay for intratumoral Top1 levels has allowed direct measurement of Top1 levels and Top1 inhibitor effects on target. Preclinical pharmacodynamic modeling of indenoisoquinoline and topotecan time and dose effects was performed in both topotecan-responsive and -nonresponsive xenografts. Topotecan-responsive A375 and HCT 116 xenografts showed higher baseline Top1 levels than nonresponsive SK-MEL-28. Top1 levels decreased in response to topotecan, with a maximal decrease after 4 to 7 hours in all xenograft models tested. Substantial biological variation in Top1 levels was observed in control animals, particularly for A375 xenografts, which may complicate use of Top1 levels as a marker of target engagement. Baseline data in the limited number of patient specimens analyzed also show diverse Top1 levels in different tumors. The total Top1 immunoassay has been transferred to the National Clinical Target Validation Laboratory, NCI (Bethesda, MD) to assess pharmacodynamic response in tumor biopsies in patient samples from current clinical trials of indenoisoquinoline Top1 inhibitors conducted at the NCI, and development of a Top1cc assay is in progress.

## Materials and Methods

### Ethics Statement

The Frederick National Laboratory for Cancer Research is accredited by Association for Assessment and Accreditation of Laboratory Animal Care International and follows the USPHS Policy for the Care and Use of Laboratory Animals. All the studies were conducted according to an approved animal care and use committee protocol in accordance with the procedures outlined in the “Guide for Care and Use of Laboratory Animals” (National Research Council; 1996; National Academy Press; Washington, D.C.).

All patients and healthy donors gave written informed consent for study inclusion and were enrolled on NCI institutional review board-approved protocols. The study was performed in accordance with the precepts established by the Helsinki Declaration. The study design and conduct complied with all applicable regulations, guidances, and local policies and was approved by the NCI institutional review board.

### Animal models

Female athymic nude (NCr) mice (Frederick National Laboratory for Cancer Research Animal Production Program) were implanted with the human melanoma cell lines A375, Colo829, and SK-MEL-28 or the human colon cancer cell line HCT 116 as previously reported [34]. All cell lines were purchased from ATCC (Manassas, VA). All mice developed tumors, and the tumors were maintained by serial in vivo passage using tumor fragment transplantation when the donor tumors reached 10 to 15 mm in diameter. Tumors were staged to a preselected size (weight  = 150–300 mg) calculated using the following formula: weight (mg)  =  (tumor length x [tumor width]^2^)/2 [45]. Mice were housed in sterile, filter-capped polycarbonate cages (Allentown Caging, Allentown, NJ), maintained in a barrier facility on a 12-hour light/dark cycle, and were provided sterilized food and water, ad libitum. Mice were randomized before initiation of treatment using a commercial software program (Study Director, Studylog Systems, Inc., San Francisco, CA).

### Drug administration

Topotecan (NSC 609699) was obtained through the Developmental Therapeutics Program, NCI, and the indenoisoquinoline NSC 724998 was initially synthesized by Dr. Mark Cushman, Purdue University and provided by the Developmental Therapeutics Program Repository, NCI. Topotecan was administered intraperitoneally in a sterile water vehicle. NSC 724998 was administered intravenously in a vehicle composed of 20 mM hydrochloric acid: 10 mM citric acid: 5% dextrose (1∶1∶6). Drugs were administered as a single dose in 0.1 mL vehicle/10 g body weight. The established single-dose MTD in mice for NSC 724998 is 25 mg/kg and for topotecan is 15 mg/kg [46]. These doses are referred to as the single-dose MTD for each compound within the manuscript; doses in this report are described as fractions of the single-dose MTD. The MTD for topotecan administered to mice once a day for 5 consecutive days (QDx5) is 4.7 mg/kg and is equitoxic to the human therapeutic dose of 1.5 mg/m^2^/day for 5 consecutive days [47], [48]. Topotecan, NSC 724998, or vehicle were administered at the times and doses indicated. In some experiments, the use of the water vehicle was omitted due to constraints caused by the number of animals available.

### Xenograft collection

Specimen collection and handling conditions tested were limited to those commonly used at the NIH Clinical Center (Bethesda, MD) and extramural sites participating in NCI clinical trials. All specimens were collected from anesthetized donors and immediately flash-frozen, dry, in pre-cooled o-ring screw-cap conical vials as previously described [34], [49]. Briefly, cohorts of at least three mice per drug dose and time point were anesthetized with isoflurane, and the xenograft tumor pieces were collected by resection, once surgical anesthesia was reached (no toe pinch). Tumor pieces were collected for initial testing of the immunoassay on biopsy extracts as previously described with tumor pieces sized to approximately 20 mg [30], [34]. Sampling was timed from the beginning of drug administration. Frozen tumor biopsies were stored in liquid nitrogen (preferred) or −80°C until processing.

### Patient blood and biopsy collection

Needle biopsies were collected from patients with cancer (various types of solid tumors) at the Center for Cancer Research, NCI, and immediately flash-frozen in liquid nitrogen or a dry ice/ethanol bath per our previously published method [34]. Blood samples from patients with cancer (various types of solid tumors) at the Center for Cancer Research, NCI and at NCI-Frederick were collected in 8-mL Cell Prep Tubes (Becton Dickinson, Rockville, MD). PBMCs were isolated, counted, and flash-frozen in liquid nitrogen or a dry ice/ethanol bath following our previously published method [34].

### Biopsy extract preparation

Frozen biopsies were resuspended in 0.5 mL lysis buffer (10 mM Tris HCl pH 8.5, 0.1% sodium dodecyl sulfate) supplemented with protease inhibitor cocktail tablets (Roche Applied Science, Indianapolis, IN) and 1 mM phenylmethanesulfonyl fluoride (Sigma-Aldrich, St. Louis, MO). PhosSTOP (Roche Applied Science, Indianapolis, IN) was added to buffer but did not affect Top1 levels (data not shown) but allowed sample extracts to be processed for additional phosphorylation markers. The excised xenograft tumor pieces were homogenized with a PRO200 homogenizer with 7 mm probe (ProScientific, Oxford, CT) in an ice bath. Patient 18-gauge needle biopsies were homogenized using an Ultrasonic Processor with microtip (Cole Parmer, Vernon Hills, IL), kept in an ice bath, and homogenized again. Samples were kept on ice throughout the processing steps. Tumor extracts were stored at −80°C and protein levels were determined with the Bicinchoninic Acid (BCA) Protein Assay Kit (Thermo Scientific Pierce, Rockford, IL) according to the manufacturer's instructions. During assay development, it was noted that storage of samples at very dilute concentrations resulted in degradation of Top1 protein (data not shown). As a result assay procedures were modified to either run diluted samples in the immunoassay the day they were processed, or to store sample extracts at a concentration of at least 1 mg/mL until the assay could be performed.

### PBMC preparation and analysis

Total protein was extracted from 1×10^7^ cells/mL in the same lysis buffer used for biopsy samples and cells were homogenized using an Ultrasonic Processor with microtip (Cole Parmer) in an ice bath as previously described [34]. PBMC extracts were stored at −80°C.

### Top1 immunoassay

The Top1 immunoassay was performed as previously described [33] with the following exception. Probe antibody and HRP-conjugate were pre-incubated with mouse serum (Sigma, 1∶1000) to lower background signal. Mouse anti-Top1 monoclonal antibody clone C21.1 (BD Biosciences Pharmingen, 1∶1000), was used as the capture antibody. Pure rTop1 (EMD Biosciences, Inc.,) was used as to make the standards. Samples and standards were diluted in PBS-casein and incubated overnight at 2°C to 8°C. Rabbit anti-Top1 polyclonal antibody Ab28432 (Abcam, 1∶500 in PBS-casein) was used as the probe followed by the addition of extra serum-absorbed goat-anti-rabbit horseradish peroxidase conjugate (KPL, 1∶1000). Finally, Pico-ELISA substrate (Thermo Scientific Pierce) was added and chemiluminescence was measured on an Infinite 200M (Tecan Group Ltd., Morrisville, NC). Top1 levels for tumor biopsy and xenograft samples were normalized to 1 µg protein load and to 1×10^7^ cells for PBMCs.

### Top1 immunoassay validation for tumor extracts

The Top1 immunoassay was validated for analytical performance in xenograft biopsies collected using clinically relevant specimen collection and preparation procedures [28]. Assay conditions, standards and controls were identical to those previously described and samples were assayed following the standard operating procedures established for determining Top1 levels in the NCI 60 cell line panel [33]. Assay controls and standards were run on each plate. For inter-day and intra-assay performance experiments, A375 tumor extract controls were made by dilution of a 2 µg/mL stock 1∶80 (Low), 1∶40 (Mid), and 1∶20 (High); MCF7 control extracts were made by dilution of 1×10^7^ cells/mL stock 1∶1250 (Low), 1∶250 (Mid), and 1∶50 (High).

In spike/recovery experiments, pure rTop1 standards (0.1 to 6.25 ng/mL) were spiked into A375 xenograft extracts with total protein loads of 0.25, 0.5 or 1.0 μg. The Top1 immunoassay was then performed to determine total recovery of spiked rTop1 standards; percent recovery was calculated based on the initial rTop1 spiked concentration. Dilution linearity was determined by performing a 1∶2 serial dilution of three A375 and three HCT 116 xenograft extracts into assay buffer (5, 2.5, 1.25, 0.625 μg total protein/well).

Intra-plate and inter-day (intra-assay) performance was performed using six xenograft tumor extracts assayed at four dilutions on three separate days by a single operator. The CV of the apparent specimen concentration based on reading the standard curve was determined. Inter-laboratory performance was determined using 24 matched samples originating from 6 HCT 116 xenograft extract samples, 3 extracts were prepared by each laboratory. Extract dilutions were prepared independently at each site for a final concentration of 5, 3.75, 2.5, and 1.25 μg/mL.

### Statistical analysis

Values for mean, median, standard deviation, correlation coefficients (R^2^), and CV were determined using Microsoft Excel software. The Grubb's test with significance level (α) set at 0.05 was used to detect outliers within drug treated cohorts with a minimum of 5 animals using GraphPad software (GraphPad Software Inc., La Jolla, CA). Student's unpaired one-tailed *t*-tests with the significance level (α) set at 0.05 (95% confidence level) were used to compare drug treatment groups to untreated or vehicle; Student's unpaired two-tailed *t*-tests were used to compare untreated or vehicle control groups to each other.

## Supporting Information

Figure S1 **Western blot analysis of Top1 levels in topotecan-responsive and –nonresponsive cell lines.** (A) Total protein extracts from A375 and SK-MEL-28 cell lines were assessed by Western blot following 7 hour treatment with either no drug or increasing concentrations of topotecan (1, 10, or 100 µM). (B) Relative intensity of Top1 bands in Western blot at IR700. (C) Total protein extracts from A375 and SK-MEL-28 cell lines treated with 100 µM topotecan for 1, 4, or 7 hours or no drug for 1 hour (zero time point). (D) Relative intensity of Top1 bands in Western blot at IR700.
(TIF)

Supporting Information S1 (DOCX)
