# Supplementary figures and images for: Development and Validation of an Immunoassay for Quantification of Topoisomerase I in Solid Tumor Tissues
Source: PLoS One. 2012 Dec 28;7(12):e50494. doi: 10.1371/journal.pone.0050494 (PMC3532478; doi:10.1371/journal.pone.0050494)

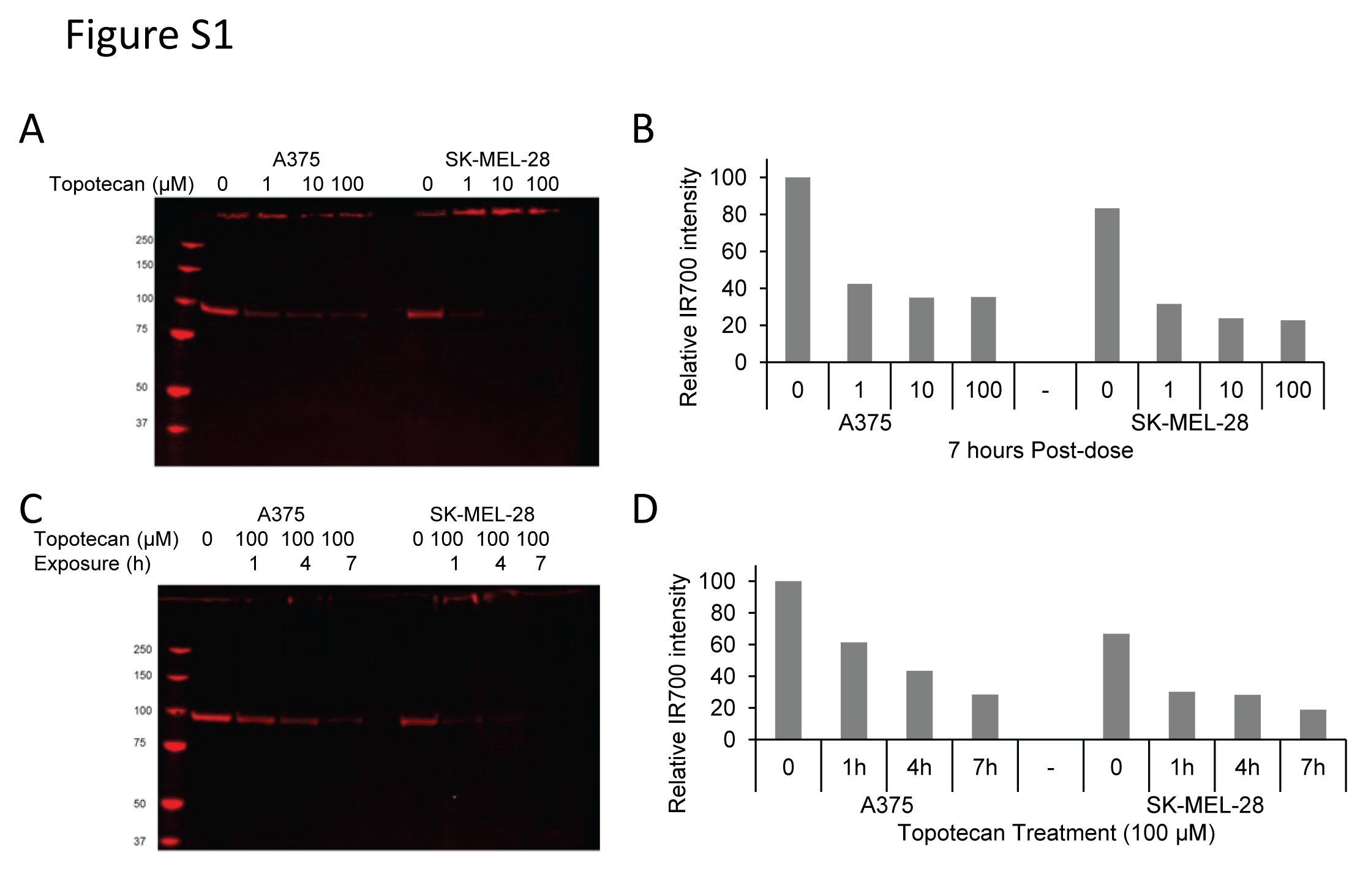

Supplement: Figure S1 — Western blot analysis of Top1 levels in topotecan-responsive and –nonresponsive cell lines. (A) Total protein extracts from A375 and SK-MEL-28 cell lines were assessed by Western blot following 7 hour treatment with either no drug or increasing concentrations of topotecan (1, 10, or 100 µM). (B) Relative intensity of Top1 bands in Western blot at IR700. (C) Total protein extracts from A375 and SK-MEL-28 cell lines treated with 100 µM topotecan for 1, 4, or 7 hours or no drug for 1 hour (zero time point). (D) Relative intensity of Top1 bands in Western blot at IR700. (TIF) [file pone.0050494.s001.tif]
