# Supplementary material for: Development and Validation of an Immunoassay for Quantification of Topoisomerase I in Solid Tumor Tissues
Source: PLoS One. 2012 Dec 28;7(12):e50494. doi: 10.1371/journal.pone.0050494 (PMC3532478; doi:10.1371/journal.pone.0050494)
Supplement: Supporting Information S1 — (DOCX) [file pone.0050494.s002.docx]

# Supporting Information

Development and Validation of an Immunoassay for Quantification of Topoisomerase I in Solid Tumor Tissues

Pfister et al.

# Results and Discussion

Western blot analysis with the mouse monoclonal antibody (clone C21.2) used as the capture antibody in the ELISA assay was performed using extracts from A375 and SK-MEL-28 cell lines (Figure S1). Untreated A375 and SK-MEL-28 cell lines had intense bands at approximately 100 kDa, corresponding to full-length Top1 (Figures S1A and S1C). The band decreased in a dose- and time- dependent manner with Top1 inhibitor treatment (Figures S1B and S1D, respectively). This is consistent with the Top1 immunoassay results observed in xenograft samples (Figures 2 and 3). It also agrees with our previously published data, where we demonstrated that Top1 levels measured by this immunoassay in extracts from cells treated in vitro with topotecan decreased in response to drug treatment [1].

# Materials and Methods

## Cell lines and drug administration

A375 and SK-MEL-28 cells (ATCC) were grown in T75 (75 cm^2^) flasks (Corning) in RPMI 1640 media (Lonza) supplemented with 10% fetal bovine serum (ATCC) and 50 mg/L gentamicin (Lonza) in an incubator at 37°C with 5% CO_2_. Cells were treated with topotecan in vitro for times and doses indicated. Topotecan (NSC 609699) was obtained through the Developmental Therapeutics Program, NCI. Cells were harvested while in exponential growth and lysed directly in the flask with lysis buffer (10 mM Tris HCl pH 7.5, 1.0% sodium dodecyl sulfate) supplemented with c0mplete protease inhibitor cocktail tablets, 1 mM phenylmethanesulfonyl fluoride, and PhosSTOP (Roche Applied Science, Indianapolis, IN). Lysates were sonicated for 10 seconds each on ice with a Sonic Dismembranator (Thermo Fisher Scientific). Protein levels were determined with the BCA Protein Assay kit (bicinchoninic acid; Thermo Scientific) using bovine serum albumin as the standard.

## Western blotting

Western blotting was performed on A375 and SK-MEL-28 cell line protein extracts (40 μg/well). Proteins were separated on 4% to 20% precast gels (Invitrogen) by SDS-PAGE, and run at 120V. The separated proteins were blotted onto nitrocellulose membranes using the iBlot system (Invitrogen). Membranes were blocked overnight with LICOR blocking buffer (LI-COR Biosciences). The blots were probed with 1:1000 mouse anti-Top1 monoclonal antibody clone C21.1 (BD Biosciences Pharmingen) or rabbit anti-Top1 polyclonal antibody Ab28432 (Abcam). Anti-mouse IR-680 or anti-rabbit IR-800 (LI-COR Bioscience) secondary antibodies were used at 1:10,000 dilution. Blots were scanned using Odyssey IR Imager (LI-COR Biosciences) and quantified using LI-COR software v.3.0.

# References

1. Pfister TD, Reinhold WC, Agama K, Gupta S, Khin SA, et al. (2009) Topoisomerase I levels in the NCI-60 cancer cell line panel determined by validated ELISA and microarray analysis and correlation with indenoisoquinoline sensitivity. Mol Cancer Ther 8: 1878-1884.
